# Traumatic Atrial Septal Defect with Tricuspid Regurgitation Following Blunt Chest Trauma Presenting as Hypoxemia: A Case Report

**DOI:** 10.5811/cpcem.20279

**Published:** 2024-09-21

**Authors:** Aamir Rashid, Farooq Ahmad Ganie, Hilal Rather, Shahood Ajaz Kakroo, Raja Suhail Shounthoo

**Affiliations:** * Sher-i-Kashmir Institute of Medical Sciences Soura, Department of Cardiology, Srinagar, Jammu and Kashmir, India; † Sher-i-Kashmir Institute of Medical Sciences Soura, Department of Cardiovascular and Thoracic Surgery, Srinagar, Jammu and Kashmir, India; ‡ Sher-i-Kashmir Institute of Medical Sciences Soura, Department of Anesthesia, Srinagar, Jammu and Kashmir, India

**Keywords:** cardiac blunt trauma, traumatic ASD, tricuspid regurgitation, hypoxia, echocardiographic examination, case report

## Abstract

**Introduction:**

Although myocardial injury is common after blunt chest trauma, tricuspid valve injury associated with traumatic atrial septal defect resulting in acute hypoxia is an infrequent event. We report an unusual case of blunt chest trauma referred to us for unexplained hypoxemia, emphasizing the unusual nature of injury and the importance of comprehensive cardiac evaluation in such cases.

**Case Report:**

A 35-year-old male presented to the emergency department after falling from a tree from an approximate height of 15 feet. He sustained multiple rib fractures and a left hemopneumothorax. Examination revealed decreased air entry over the left hemithorax and a systolic murmur over the left sternal border. Electrocardiography showed a junctional rhythm, and troponin levels were significantly elevated. Despite tube thoracostomy, the patient remained hypoxemic. Cardiology evaluation revealed a flail tricuspid valve with severe regurgitation and a traumatic atrial septal defect (ASD). Bidirectional shunting across the atrial septal defect was causing hypoxemia. The patient underwent surgical repair of the ASD and tricuspid valve, which resulted in a successful outcome.

**Conclusion:**

Our case highlights the need for comprehensive cardiac evaluation in such patients. In addition to sonography for trauma, point-of-care echocardiographic examination should be a part of the focused assessment.

## INTRODUCTION

Traumatic injuries result in close to six million fatalities worldwide annually.^1^ Thoracic injuries account for the highest morbidity and mortality among trauma patients. Up to 10–25% of all traumatic fatalities involve cardiac or aortic injuries.^1^ The right ventricle (RV) and right atrium (RA) are more commonly involved than the left ventricle and left atrium, due to their anatomical location and wall thickness. The RV and RA are positioned anteriorly in the chest, making them more susceptible to direct trauma. Additionally, the walls of the RV and RA are thinner compared to the thicker, more muscular walls of the left ventricle, making them more vulnerable to injury upon impact.^1^

Valve injury secondary to blunt trauma is uncommon. Ventricular septal defects are extremely rare, and atrial septal defects (ASD) are even less frequent, with only a few case reports of traumatic ASDs.^2^ While acute traumatic right-to-left cardiac shunts have been described following penetrating trauma,^2^ their occurrence following blunt chest trauma is exceedingly rare.^3^ We describe the case of a young male referred to us for unexplained refractory hypoxemia following blunt chest trauma. Our case highlights a traumatic blunt chest injury resulting in an unusual presentation.

## CASE REPORT

**Image 1:**
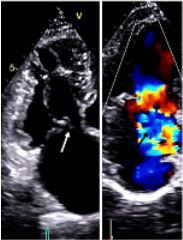
Transthoracic echocardiography showing severe tricuspid regurgitation with flail anterior leaflet of tricuspid valve. White arrow showing flail anterior leaflet of tricuspid valve and black arrow showing color Doppler of severe tricuspid regurgitation.

A 35-year-old male presented to our emergency department following a fall from a tree. He had no significant past history. He suffered multiple rib fractures and left hemopneumothorax. The physical examination showed decreased air entry over the left hemithorax and a systolic murmur over the left sternal border. Electrocardiography showed a junctional rhythm. The troponin levels were significantly increased to 56 nanograms per milliliter (ng/mL) (reference 0–1 ng/mL). The patient underwent a tube thoracostomy; however, he continued to be hypoxemic despite high-flow oxygen and appropriate management of his hemopneumothorax. Cardiology consultation was sought, and echocardiography demonstrated a flail tricuspid valve due to a rupture of the sub-valvular segment (chordal rupture) of the anterior tricuspid leaflet, resulting in severe tricuspid regurgitation (TR) (Image 1).

**Image 2:**
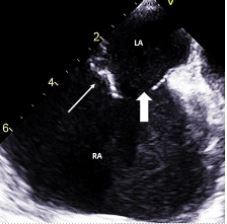
Transesophageal view at 140° showing ostium secondum atrial septal defect with flimsy torn atrial septum. White thin arrow showing flimsy torn atrial septum and white thick arrow showing atrial septal defect. *LA*, left atrium; *RA*, right atrium.

The echocardiography further showed a significant ASD with traumatic flail margins (Image 2) and a TR jet directing flow across the ASD, resulting in bidirectional shunting across the ASD, explaining the hypoxemia.

**CPC-EM Capsule**

What do we already know about this clinical entity?

*Although blunt myocardial injury is common, tricuspid valve injury associated with traumatic atrial septal defect is extremely rare.*

What makes this presentation of disease reportable?

*This case highlights a rare combination of traumatic tricuspid regurgitation and atrial septal defect leading to unexplained hypoxemia.*

What is the major learning point?

*Comprehensive cardiac evaluation, including echocardiography, is crucial in patients with blunt chest trauma and unexplained hypoxemia.*

How might this improve emergency medicine practice?

*Routine use of point-of-care echocardiography in blunt chest trauma can detect rare cardiac injuries, improving diagnosis and outcomes.*

Considering the flail traumatic margins of the ASD and the non-dilatation of the pulmonary arteries, the ASD was assumed to be traumatic rather than congenital. The patient was urgently referred to surgery and underwent a successful ASD closure with tricuspid valve repair on the second day of hospital admission.

## DISCUSSION

Our case underscores the importance of dedicated cardiac evaluation in patients with blunt chest trauma who present with unexplained hypoxemia. Although blunt myocardial injury is common, tricuspid valve injury associated with traumatic ASD is extremely rare. Valve injury secondary to blunt cardiac trauma is uncommon.^4^ The aortic valve is the most commonly involved cardiac valve following trauma, followed by the mitral and the tricuspid.^5^ Traumatic TR is usually well tolerated. However, in our patient, the presence of an associated ASD resulted in a right-to-left shunt causing severe hypoxemia.

Traumatic TR can be caused by compression and decompression of the thorax. The resulting increase in RV pressure, especially if it occurs during the isovolumic contraction phase when all valves are closed, increases the risk of injury to the atrioventricular valves with rupture of the chordae or papillary muscle from the sudden increase in intracardiac pressure.^4^ The aortic and pulmonary valves are more susceptible to injury in diastole. The mechanisms of valve injury include papillary muscle rupture, chordal tear, leaflet injury, and delayed papillary muscle necrosis secondary to contusion.^4^ Our patient suffered from a chordal tear of the anterior tricuspid leaflet and papillary muscle rupture. Tricuspid valve rupture with varying degrees of heart block has been described.^6^ Our patient had a junctional rhythm, which improved spontaneously two weeks after surgical repair.

Although traumatic ventricular septal defect has been reported to have a prevalence of 5% following blunt thorax trauma, the occurrence of traumatic ASD is rare.^7^ Getz et al^8^ postulated different mechanisms of injury in cardiac chamber rupture. These include a direct blow to the chest, indirect trauma due to a crush injury of the abdomen with a resultant increase in intracardiac pressure, bidirectional force from compression of the heart between the sternum and vertebral bodies, acceleration and deceleration injury, blast forces, and penetrating trauma. Our patient had a direct blow to the chest as a result of a fall from a height.

The atrial septum is most susceptible to injury in late systole when the atria are full and all valves are closed. In contrast, the ventricular septum is more susceptible to injury in early systole and late diastole.^8^ Delayed perforation can also occur secondary to inflammatory reaction due to septal contusion.9 The right-to-left shunt due to the severe TR jet across the ASD caused the hypoxemia in our patient. The selective streaming of the severe TR jet across the ASD was thought to be responsible for central cyanosis and has been described previously.^2^,^3^ Furthermore, acute TR can increase the RA pressure to more than the left atrium pressure, resulting in a right-to-left shunt across the ASD. The right-to-left shunt has been described in patients with congenital ASD and TR.^10^

## CONCLUSION

The patient described sustained a traumatic Tricuspid valve injury resulting in severe TR and an ASD with shunting resulting in hypoxemia after a fall from height. This case highlights the need for comprehensive cardiac evaluation in blunt chest trauma. Point-of-care echocardiographic examination should be an essential part of focused assessment along with sonography for cardiac blunt trauma.
